# Anti-cancer effects of 3,5-dimethylaminophenol in A549 lung cancer cells

**DOI:** 10.1371/journal.pone.0205249

**Published:** 2018-10-11

**Authors:** Pei-Ying Lin, Yu-Jung Chang, Yu-Chen Chen, Chin-Hung Lin, Pinar Erkekoglu, Ming-Wei Chao, Chia-Yi Tseng

**Affiliations:** 1 Department of Bioscience Technology, College of Science, Chung Yuan Christian University, Zhongli district, Taoyuan, Taiwan; 2 Department of Radiology, Taoyuan General Hospital, Taoyuan district, Taoyuan, Taiwan; 3 Hacettepe University, Faculty of Pharmacy, Department of Toxicology,Ankara, Turkey; 4 Center of Nanotechnology, Chung Yuan Christian University, Zhongli district, Taoyuan, Taiwan; 5 Department of Biomedical Engineering, College of Engineering, Chung Yuan Christian University, Zhongli district, Taoyuan, Taiwan; Institute of Biochemistry and Biotechnology, TAIWAN

## Abstract

Exposure to 3,5-dimethylaminophenol (3,5-DMAP), the metabolite of the 3-5-dimethylaniline, was shown to cause high levels of oxidative stress in different cells. The aim of the present work was to observe whether this metabolite can lead to cytotoxicity, oxidative stress, DNA damage and cell cycle changes in non-small cell lung cancer A549 cells. 3,5-DMAP caused a dose-dependent increase in cytotoxicity, generation of superoxide (O_2_^-.^), inductions in the enzyme activities orchestrating cellular antioxidant balance, increases in lipid peroxidation as well as DNA damage. However, 3,5-DMAP showed significantly lower cytotoxicity towards human lung fibroblast (HLF) cells. 3,5-DMAP also led to molecular events, like inducing apoptotic markers (ie. p53, Bad, Bax and cytochrome c); decreasing anti-apoptotic proteins (Bcl-2) and alterations in cell cycle. Our findings indicate that the cytotoxicity caused by this particular alkylaniline metabolite led to initiation of caspase 3-mediated apoptosis. Furthermore, 3,5-DMAP attenuated carcinogenic properties like migration capacity of A549 cells and eventually inhibited growth of A549 cells in an *in vivo* mouse model. Tumor sections showed that 3,5-DMAP down-regulated c-Myc expression but up-regulated p53 and cytochrome c, all of which might result in tumor growth arrest. Co-treatment with N-acetylcysteine provided reductions in cytotoxicity and positively modulated genetic events induced by 3,5-DMAP in A549 cells. In conclusion, our findings demonstrate 3,5-DMAP may be a potential anti-cancer drug in cancer, due to its self redox cycling properties.

## 1. Introduction

Approximately 10,000 new lung cancer cases occur each year, and 7000 people annually die from lung cancer in Taiwan [1]. The incidence of lung cancer is greater than combined incidences of colorectal, cervical, breast, prostate, and stomach cancers throughout the globe. The number of cases continue to grow rapidly each year [2–4]. Early symptoms of this particular cancer are not always obvious [5–8].

According to the Department of Health Statistics (Taiwan) passive smoking, hot tar fumes, radiation, asbestos, factory smokes, soot, fine suspended particles, and dust storms are the primary causes of lung cancer [2–8]. Lung cancers are classified as small cell or non-small cell carcinomas due to their consisting from different cell types (non-epithelial or epithelial-derived), respectively [9]. Small cell carcinomas are highly malignant and can easily metastasize [10]. Chemotherapy is used to treat small cell carcinoma [10–12]. Non-small cell cancer can be divided into squamous cell carcinoma, adenocarcinoma, large cell carcinoma, glandular squamous cell carcinoma, carcinoid tumors, and bronchial adenocarcinoma [9, 13, 14]. Treatments for these types of cancers primarily involve surgical excision supplemented by radiation or chemotherapy [15, 16]. However, the longer the chemotherapy administration continues, the stronger resistance is developed by cancerous cells [17, 18]. Although this treatment method may provide partial or full recovery, it also increases the risk for concurrent diseases [18]. Thus, high efficancy of an anti-cancer drug is the most priority goal in this field.

Alkylanilines are a group of chemicals. These chemicals are classified in the general chemical group “monocyclic aromatic amines” and also under the sub-group of “alkylanilines”. These chemicals are present in the environment as well as in cigarette smoke [19]. 3,5-dimethyaminophenol (3,5-DMAP) is the main metabolite of 3,5-dimethylaniline (3,5-DMA), which is one of the most abundant alkylanilines in the environment. 3,5-DMA is used in the production of different industrial chemicals (azo dyes, pharmaceuticals, detergents, wood preservatives, textiles, metal complexes and antiozonants). 3,5-DMA has also been detected in cigarette smoke [19].

Several potentially damaging species (often termed as reactive oxygen species, ROS) arise as by-products of normal metabolism or from exposure to environmental chemicals [20]. Increases in cellular ROS may lead to lipid peroxidation, which may lead to massive protein oxidation and degradation. However, protein oxidation can arise independent from lipid peroxidation after exposure to high amounts of ROS [21, 22]. ROS are also involved in a variety of different cellular processes ranging from apoptosis and necrosis to cell proliferation and carcinogenesis [23].

Recently, Chao et al. (2014) have conducted experiments using Chinese hamster ovary (CHO) cells, revealing an alternative mechanism for cytotoxic and genotoxic effects of 3,5-DMAP [24, 25]. Ye et al. (2012) suggested that 3,5-DMAP could lead to redox cycling through the corresponding quinone imines to generate ROS. The electrophilic quinoneimine intermediate metabolite, 3,5-dimethylquinoneimine (3,5-DMQI), can react with protein thiols *in vitro* [26]. Although it was first suggested that phenolic metabolites of the anilines, particularly by 3,5-DMAP, caused covalent DNA adducts and this was the underlying toxicity mechanism, high intracellular ROS production seems to be the predominant toxicity mechanism of these compounds [26]. Furthermore, this particular alkylaniline can lead to epigenetic changes by altering the acetylation of histone H3 and H4 [27]. It is a fact that high intracellular ROS production can lead to DNA damage. It was suggested that 3,5-DMAP caused high levels of intracellular ROS in different cellular fractions and might also lead to DNA single-strand damage as evidenced by Erkekoglu et al. (2014) [27]. Moreover, both genetic and epigenetic alterations caused by 3,5-DMAP further led to cell cycle G1 arrest and apoptosis [28].

Currently, there is considerable interest in using 3,5-DMAP as the drug/drug precursor against lung cancer, due to its high cytotoxic potential. Apropos to this knowledge and information, this study was designed to investigate the anticancer effects of 3,5-DMAP on cytotoxicity, antioxidant parameters, cell cycle arrest, apoptosis and cell migration in lung cancer A549 cells. Moreover, we investigated the protective effects of N-acetylcysteine (NAC) against the toxicity of 3,5-DMAP.

## 2. Material and methods

### *2*.*1* Cell line

The lung cancer A549 cells were obtained from Bioresource Collection and Research Centre (BCRC, Taiwan) and human lung fibroblast (HLF) cells, Wi-38, were obtained from ATCC (Rockville, MD, USA). Cells were cultured in Ham’s F-12K medium (Sigma-Aldrich, St. Louis, MO, USA) with 10% fetal bovine serum (FBS) (Gibco, Waltham, MA, USA) at 37°C, in a humidified atmosphere containing 5% CO_2_. For the experiments, A549 cells were treated with 3,5-DMAP in serum-free medium for one hour followed by additional 24 hours for the recovery in culture medium.

### *2*.*2* Intracellular *ROS* and superoxide (O_2_^.-^) detection assay

Intracellular ROS detection studies were performed by using a ROS detection kit (Invitrogen, Waltham, MA, USA). Cells were washed with phosphate buffered saline (PBS) and treated with fresh serum-free medium containing chloromethyl derivative of fluorescent probe 2’,7’-dichlorodihydrofluorescein diacetate (H_2_DCF-DA), namely CM-H_2_DCFDA (final concentration was 25 μM). Samples were then measured immediately with using microreader (Madison, WI). ROS levels (generated by 2.5×10^4^ viable treated cells) were expressed as the fold change of ROS produced by an equal number of viable negative control cells. Cells producing ROS were imaged by epifluorescence microscopy at 400X magnification (excitation at 488 nm, Olympus Microscope). O_2_^.-^ was detected by MitoSOX Red indicator (Invitrogen, Waltham, MA, USA). Cells were washed with Hank's Balanced Salt Solution (HBSS) (with Ca^2+^ and Mg^2+^) and treated with 5 μM MitoSOX Red reagent for 10 min in dark. Samples were measured at 510 nm by microreader. The levels of O_2_^.-^ was normalized and presented as the fold change of the control. Cells producing O_2_^.-^ were imaged by epifluorescence microscopy at 400X magnification (λ_excitation_: 596 nm, by Olympus Microscope) after treatment with the same MitoSOX protocol.

### *2*.*3* Western blotting

Total cell lysate proteins were extracted and collected after the treatments. The protein concentration was detected by using the BCA assay (Thermo Fisher Scientific, Waltham, MA, USA). After the determination of total protein concentrations, the samples were denatured and loaded (40 μg/well) onto sodium dodecyl sulfate (SDS) polyacrylamide gels (Bio-Rad, Hercules, CA, USA) for electrophoresis. Proteins were transferred to polyvinylidene difluoride (PVDF) (Bio-Rad, Hercules, CA, USA) membrane by using electrophoretic transfer (Bio-Rad, Richmond, USA). Nonspecific reactivity was blocked for 1 h at room temperature with standard 1X tris-buffered saline—Tween 20 (TBST) (Sigma-Aldrich, St. Louis, MO, USA) buffer containing 3% bovine serum albumin (BSA) (Sigma-Aldrich, St. Louis, MO, USA) and 0.02% sodium azide. Primary antibodies of superoxide dismutase 1 (SOD-1), catalase, p53, Bad, Bax, Bcl-2, cytochrome c and glyceraldehyde-3-phosphate dehydrogenase (GAPDH) were probed and visualized on Li-COR C-digit Blot scanner (LI-COR Biosciences, Lincoln, Nebraska, USA) by using chemiluminescent reagent containing luminol.

### *2*.*4* Quantification of lipid peroxidation

Lipid peroxidation in A549 cell protein extracts was quantified measuring the concentration of TBARS by a spectrofluorometric assay using a TBARS assay kit (Sigma-Aldrich, St. Louis, MO, USA) as described by Richard et al. (1992) [29]. Quantification was achieved by parallel measurements of a standard curve of known malondialdehyde (MDA) concentrations, and results were as nmol/mg protein.

### *2*.*5* Cell viability assay

Cell viability was detected using a commercial MTS assay (Promega, Fitchburg, WI, USA). The assay relies on the measurement of mitochondrial succinate dehydrogenase activity *via* conversion of MTS and phenazinemethosulfate to formazan. After treatment, a mixture of 10 μL water-soluble kit reagent plus 190 μL fresh medium was added to each well for 1 h incubation at 37°C in the dark. Supernatants (100 μL/well) were collected and the absorbance of the generated formazan was measured at 490 nm with an ELISA microreader.

### *2*.*6* Alkaline comet assay

The alkaline comet assay was used to detect both double and single strand breaks in DNA. After 24 h treatment, 50 μL of A549 cells (10^5^ cells/mL) were pipetted on agarose-coated slides. Later, the slide was covered with 1% low melting point agarose. After 2 h of lysis, the Comet slides were placed into a chamber filled with alkaline unwinding buffer (0.2 M NaOH and 1 mM EDTA) for 20 min at room temperature. Slides were laid in the electrophoresis buffer at 4°C and electrophoresis was performed for 30 min at 1 V/cm, with a current of 300 mA. After electrophoresis, the Comet slides were stained with SYBR Green (Sigma-Aldrich, St. Louis, MO, USA), according to the manufacturer’s instructions for the fluorescence imaging. Images were captured using an Olympus IX51 upright microscope, coupled with an automatic scanning stage and analyzed using software Image J. The results generated by the software showed percentage of tail DNA, which represented the level of DNA damage and Olive tail moment (OTM, a product of comet length and tail intensity). The cells treated with 100 μM H_2_O_2_ were used as the positive control. In each treatment group, 100–150 comet images were collected and analyzed. Experiments were repeated for three times and the mean of three independent experiments were given as results.

### *2*.*7* Determination of apoptosis and cell cycle

A commercial Annexin V-FITC apoptosis detection kit (Sigma-Aldrich, St. Louis, MO, USA) was used to detect the ratio of apoptosis and necrosis. After the treatments, cells were collected and stained the Annexin V-FITC/propidium iodide (PI) reagents according to the manufacturer’s instructions. Stained cells were determined by FACScan flow cytometer (Becton Dickinson, CA) and analyzed with using ModFit software (Becton Dickinson, CA). The apoptotic and necrotic cells were presented as the percentage of total cell numbers. For determination of cell cycle arrest, the exponential growing A549 cells were treated with 3,5-DMAP at the indicated doses. The cells were permeabilized in 100% cold ethanol at -20°C for one day. After washing twice with cold 10% FBS, cells were centrifuged at 500 *× g* to remove debris. After washing with PBS, the cells were stained with propidium iodide (PI) solution (15 mg/ml PI and 2.5 mg/ml RNase A) (Sigma-Aldrich, St. Louis, MO, USA) and cell cycle phases were determined by FACScan flow cytometer (Becton Dickinson, CA). The cell cycle phase levels were presented as the percentage of total cell cycle phases (G1+S+G2/M).

### *2*.*8 Caspase 3/7* activity assay

Caspase activity was assessed using a commercial Caspase-Glo 3/7 assay kit (Sigma-Aldrich, St. Louis, MO, USA). After treatment, the plates were removed from the incubator and allowed to equilibrate to room temperature for approximately 30 min. Later, caspase-Glo 3/7 assay reagent (200 μL/well) was applied according to the manufacturer’s instructions. Relative Luminance Unit (RLU) emitted by the product was measured using Promega microreader (Promega, Fitchburg, WI, USA).

### *2*.*9* Cell migration assay

The cell migration was measured in the Corning Transwell-Clear 24-well insert plates (Corning, Corning, NY, USA) using Polyester (PET) Membrane (8-μm pore size) Transwell-Clear Inserts (Corning, Corning, NY, USA). 3,5-DMAP-treated A549 cells (2.5×10^4^) were suspended in 0.1 mL serum-free medium, added to the upper compartment and 0.6 mL of the medium (with 10% FBS) was added to the lower compartment. After 12 h additional incubation, the A549 cells on the lower surface of PET membrane were fixed with 4% paraformaldehyde and stained with 2% crystal violet (Sigma-Aldrich, St. Louis, MO, USA). The cells were visualized with using a light microscope at 200x magnification. Migration ability of A549 cells were quantified by counting the cells that migrated to the lower side of the PET membrane with using Image J software. The migration ratio was generated by dividing the number of viable cells to the amount of control cells in the assessed region and results were presented as the fold change of the control.

### *2.10* Tumorigenicity testing

The Institutional Animal Care and Use Committee of Chung Yuan Christian University approved the animal experiment protocol. Four week-old female nude mice were obtained from LASCo Laboratory (Taipei, Taiwan) in specific pathogen-free conditions.

1. Group 1:A549 cells were injected for tumor formation as control.
2. Group 2: A549 cells were pretreated *in vitro* with 3,5-DMAP in two doses of 25 μM for 24 h.
3. Group 3: A549 cells were pretreated *in vitro* with 3,5-DMAP in two doses of 25 μM 50 μM for 24 h.
4. In group 4, A549 cells were pretreated in vitro with 50 μM cisplatin for 24 h.

After wash with PBS, the tumorigenicity of cells was tested by injecting 2×10^6^ cells in 0.2 ml PBS per site subcutaneously (s.c.) into the dorsal flank of nude mice. Within the following 32 days, the volume of tumor and the weight of nude mice were examined every two days. Growth inhibition (GI), expressed as a percentage of control, was then calculated by the following equation which takes into account both the tumor volume of treated compared to untreated cells as well as the fraction of injected sites that grew tumors:

$$\begin{array}{c} \%\;GI:(1-\frac{Α}{Β})\times 100\%\\ \mathrm{GI}:\;\mathrm{growth}\;\mathrm{inhibition}\\ A:\;\mathrm{tumor}\;\mathrm{volume}\\ B:\;\mathrm{tumor}\;\mathrm{volume}\;\mathrm{in}\;\mathrm{positive}\;\mathrm{control} \end{array}$$

GI: growth inhibition

A: tumor volume

B: tumor volume in positive control

### *2.11* Hematoxylin & Eosin stain and immunofluorescence stain

Tumors were perfused and fixed in 4% paraformaldehyde (PFA). The tumors were embedded in FSC 22 Frozen section media (Leica, Germany) and soaked in liquid nitrogen. Frozen sections were prepared at 4 μm thickness and fixed by 4% PFA. The sections were stained with hematoxylin (Sigma-Aldrich, St. Louis, MO, USA) for 5 min and washed out with distilled water and 95% ethanol. Eosin was then applied for 3 min. Sequential dehydration steps (50 to 100% ethanol and xylene) were followed and mounting solution was applied. For immunofluorescence staining, the sections were heated for antigen retrieval and then blocked with blocking buffer overnight. The sections were incubated with rabbit polyclonal primary antibodies anti-c-Myc (1:200) (Abcam, Cambridge, MA, USA), anti-cytochrome c (1:200) (Abcam, Cambridge, MA, USA) and mouse monoclonal primary antibodies anti-p53 (1:400) (Abcam, Cambridge, MA, USA) 24 h. Then, the sections were incubated with secondary antibodies labeled Alexa 488-conjugated goat anti-rabbit IgG (Invitrogen, Waltham, MA, USA)for 1 h. The sections were mounted with Fluoromount-G^TM^ with DAPI (eBioscience, Waltham, MA, USA) and observed on an Olympus IX51 fluorescent microscope.

### *2.12* Statistical analysis

For statistical analysis, GraphPad statistics software was used. Each experiment was performed in triplicate and repeated three times. The results were expressed as mean±SD for three independent experiments and the differences between the groups were determined by Kruskal-Wallis followed by Mann Whitney U test. *, ** and *** indicate p<0.05, p<0.01 and p<0.001 significant difference from the control, respectively.

## 3. Results and discussion

### *3*.*1* Higher cytotoxic property in *A549* of *3*,*5-DMAP* than HLF

**Fig 1:**
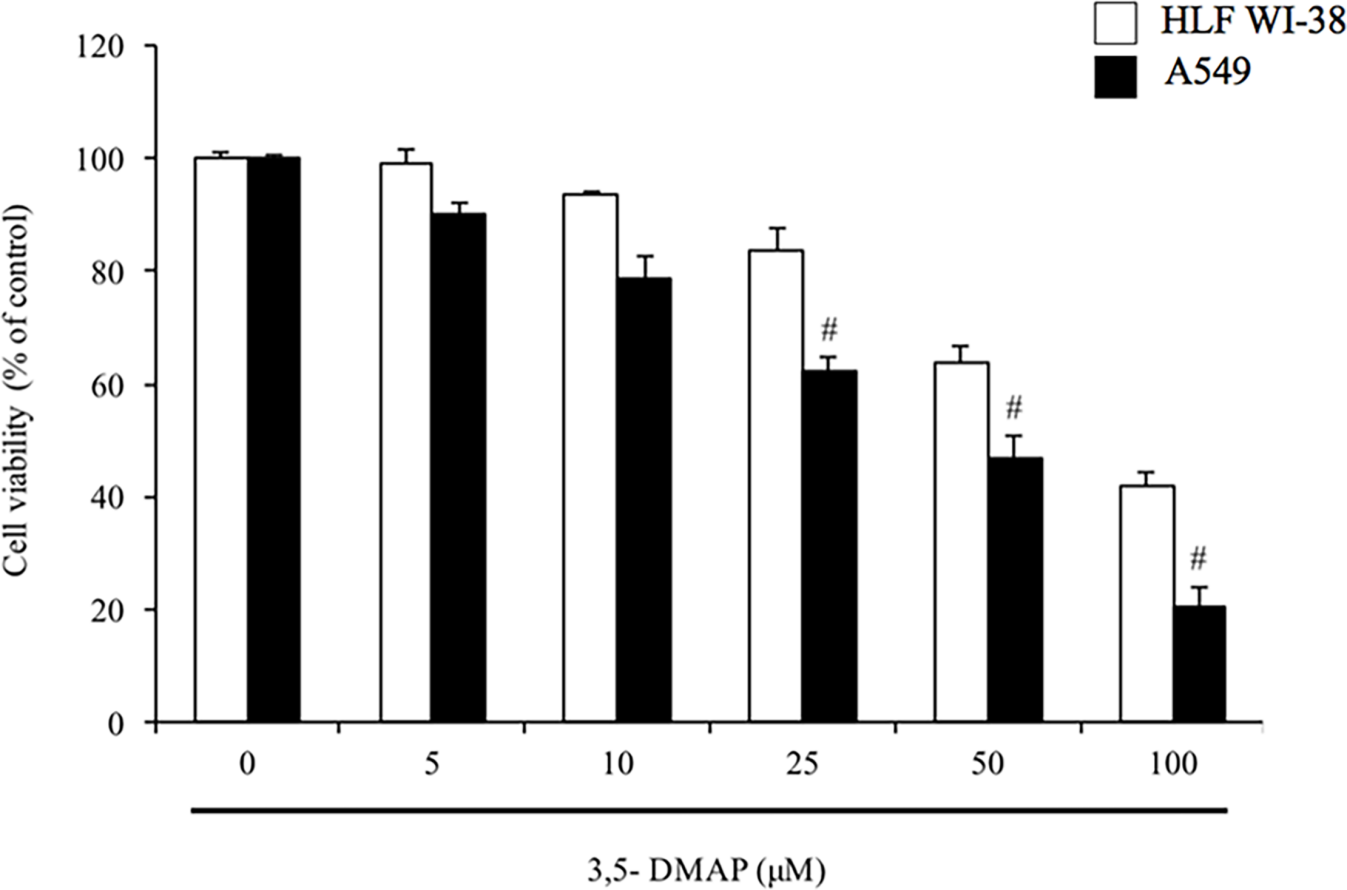
Cytotoxic effects of *3*,*5-DMAP* in *A549* lung cells and *HLF* cells at different concentrations by using MTS assay. 3,5-DMAP showed higher cytotoxic property in A549 lung cells when compared to HLF cells. # shows p<0.05 compared with 3,5-DMAP-treated A549 samples.

We used the MTS test to detect the effects 3,5-DMAP had on the viability of human lung fibroblast HLF cells, and A549 lung cancer cells. As shown in Fig 1, we found that the vitality of both cells were reduced as the 3,5-DMAP dosage increased, but cytotoxicity was not observed in HLF cells at the doses <25 μM. At different concentrations, A549 lung cells and HLF cells had different cell viabilities: After administering 25 μM of 3,5-DMAP, HLF cell showed 83% cell viability while A549 cells only had 62% cell viability. With the treatment of 50 μM of 3,5-DMAP, HLF showed 64% cell viability while A549 lung cells only showed 47% viability. Moreover, after applying 100 μM of 3,5-DMAP to both of the cell types, HLF showed two-fold higher viability (42%) when compared to A549 lung cells (21%). The median inhibitory concentrations (IC_50_) for both cells were achieved at 75 μM of 3,5-DMAP for HLF cells and 45 μM for A549 cells. Obviously, 3,5-DMAP did not produce significant cytotoxicity to human lung fibroblasts even at very high concentrations; however showed higher cytotoxic effect on A549 lung cancer cells at the same concentrations. Although the mechanism of 3,5-DMAP’s lower cytotoxicity towards non-cancerous cells remains unknown, these results mean that 3,5-DMAP can efficiently kill A549 lung cancer cells without harming normal cells especially at 25 μM, which was the treatment dose chosen for the further experiments in this study.

### *3*.*2 ROS* induction by *3*,*5-DMAP* causes lipid peroxidation, cytotoxicity and *DNA* damage in *A549* cells

Almost 80% of the lung cancer deaths is suggested to be caused by cigarette smoking. Among all the chemicals in cigarette smoke, polycyclic aromatic hydrocarbons (PAHs) and alkylanilines are considered to be one of the most important chemicals that may lead to carcinogenesis [30, 31]. Alkylanilines are a class of chemicals that may affect human health without well-clarified genotoxicity mechanism/s [32]. 3,5-DMA is present in cigarette smoke and therefore whole population is exposed to this chemical by smoking or secondhand smoke.

**Fig 2:**
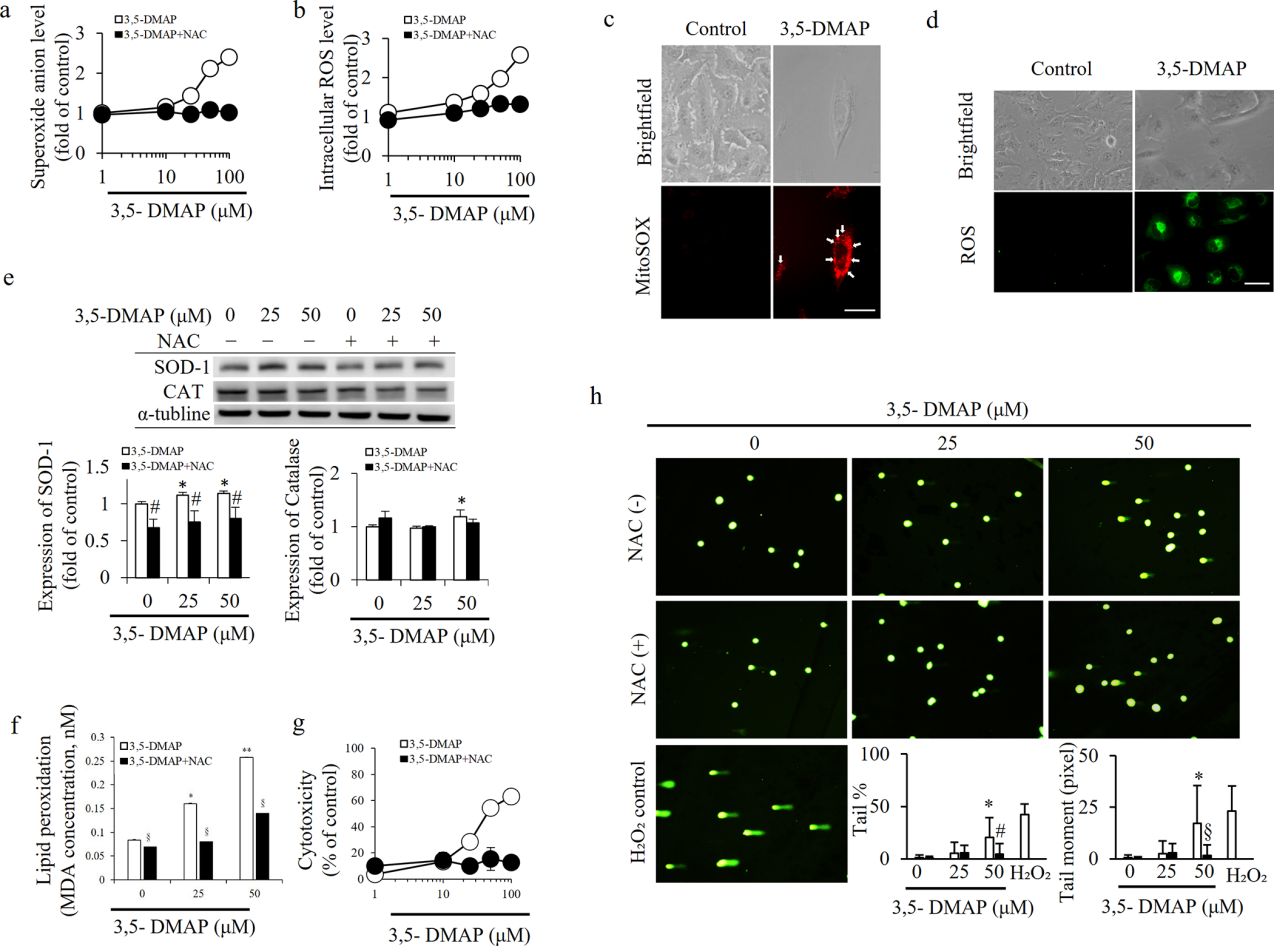
3,5-DMAP-induced high intracellular O_2_^.-^ and ROS production caused lipid peroxidation, cytotoxicity and DNA damage in A549 cells. (A) Intracellular O_2_^.-^ levels were measured by MitoSOX kit. (B) The ROS level was measured by CM-H_2_DCFDA ROS detection kit. (C) O_2_^.-^ and (D) ROS images of A549 cells treated with 3,5-DMAP. Magnification = 400X. Scale bar = 20 μm. Arrow heads point out O_2_^.-^ spots. (E) Western blot suggests that 3,5-DMAP-induced ROS in turn induces SOD-1 and CAT expressions in A549 cells. (F) Lipid peroxidation was detected using MDA assay kit. (G) MTS assay detected the cytotoxicity in response to 3,5-DMAP exposures. The data was normalized and presented as percentage of the control. (H) Cells were treated with 0, 25 and 50 μM 3,5-DMAP, 50–150 comets were collected and analyzed for each experiment. Fluorescence microscopy magnification = 400X. Scale bar = 20 μm. % tail DNA and tail moment (a function of the distance and intensity of DNA from the center of the comet head) are quantified and significant DNA damage was detected after treatment with two different doses of 3,5-DMAP (25 and 50 μM). # and § shows p<0.05 and p<0.01 compared with 3,5-DMAP-treated samples.

High ROS production was addressed as the predominant mechanism for the cytotoxic and genotoxic effects of alkylanilines in CHO cells [25, 26]. Our previous findings suggested that 3,5-DMAP, the main metabolite of 3,5-DMA, could cause cytotoxicity in a dose-dependent manner, possibly due to its ability to mainly generate oxidative stress [25, 27]. 3,5-DMAP can generate ROS through a redox cycle in which it is biotransformed to its corresponding quinone imines [24]. The electrophilic quinoneimine intermediate, 3,5-DMQI, mainly reacts with protein thiols [26]. In addition, Chao et al. (2014) indicated that ROS production remained elevated for up to 5 days following the one-hour treatment with 3,5-DMAP and this particular alkylaniline metabolite mainly caused the induction of O_2_^.-^ generation in CHO cells [24]. The current results also indicate that 3,5-DMAP converts to 3,5-DMQI and indeed causes O_2_^.-^ byproduct production in A549 cells (Fig 2A). In the present study, we also determined that A549 cells had high levels of intracellular ROS and O_2_^.-^ following 3,5-DMAP exposure (Fig 2B). Sequentially, O_2_^.-^ reacts with H_2_O and generates intracellular oxidative stress in A549 lung cells. Accordingly, intracellular localization of O_2_^.-^ (Fig 2C) and ROS (Fig 2D) were investigated first by imaging cells with epifluorescence microscopy after exposure to 50 μM of 3,5-DMAP. Fig 2C shows data after 24 h after A459 cells were exposed to 3,5-DMAP for 1 h. Almost none of control cells showed evidence of O_2_^.-^ production. After exposure to 50 μM 3,5-DMAP, 80% of cells were fluorescent, indicating presence of oxidative stress (Fig 2D). O_2_^.-^ is observed in the cytoplasm and total ROS production also occurs in the nucleus as well. As shown in Fig 2E, O_2_^.-^ levels increased up to 2.5 fold of control at 100 μM of 3,5-DMAP and only 1.5 fold of increase after co-treatment with NAC (5 mM) Therefore, we have considerable interest in using 3,5-DMAP to investigate its anticancer effects.

Western blot analyses were performed to show how SOD-1 and CAT expressions were affected from exposure to 3,5-DMAP at both 25 μM and 50 μM. Moreover, the modifying effect of NAC was also assessed (Fig 2E). It is well-known the antioxidant enzyme SOD catalyzes the dismutation of O_2_^.-^ into O_2_ and H_2_O_2_ [33]. After A549 cells were exposed to 3,5-DMAP for 1 h at different concentrations, there was a dose dependent increase in the expression of SOD-1 after 24 h. This response was completely blocked by NAC (5 mM) along with 3,5-DMAP at both of the exposure concentrations (25 μM and 50 μM). The blockage may be an indicator of reduced intracellular oxidative stress supplied by NAC and therefore, the expression of SOD-1 was decreased concordantly. Previously, we observed that SOD activity significantly increased in 3,5-DMAP treated CHO AS52 cells and AA8 cells [27, 28].

CAT is another important antioxidant enzyme that converts H_2_O_2_ to H_2_O and O_2_ [34]. CAT expression was higher than control after treatment of A549 cells with both 25 μM and 50 μM of 3,5-DMAP. NAC co-treatment with 50 μM of 3,5-DMAP reduced CAT expression almost to control levels. In 3,5-DMAP treated CHO AS52 cells and AA8 cells, CAT activity was lower when compared to controls [27, 28]. This phenomenon has two explanations: i. there may be some post-translational changes in CAT protein and these changes may result in decreases in its activity; ii. CAT expression and/or activity are regulated differently in A549 and CHO cells. Although interpreting the data obtained from the current work is hard due to the complexity of intracellular redox processes, we can suggest that high expressions of both SOD and CAT (only at 50 μM of 3,5-DMAP) enables the cell to keep its oxidant/antioxidant balance when exposed to an oxidant insult.

As an index of lipid peroxidation, MDA levels were measured. As shown in Fig 2F, ROS induction by 3,5-DMAP caused lipid peroxidation in a dose dependent manner. The high ROS levels caused by 3,5-DMAP in A549 cells was significantly mitigated by the antioxidant ability of NAC. This suggests that 3,5-DMAP induces lipid peroxidation which might lead to high cellular toxicity in A549 cancer cells. Previously, we have also observed that lipid peroxidation increased in CHO AS52 cells and AA8 cells, after exposure to 25 μM of 3,5-DMAP in both cytoplasm and nucleus. Moreover, in these cells, protein oxidation levels also increased after exposure to 25 μM of 3,5-DMAP; possibly due to high levels of lipid peroxidation which is suggested to be one of the triggering factors of protein oxidation [27].

As shown in Fig 2H, we used the Comet assay to assess the influence of ROS generation on reflected in DNA strand breaks in A549 cancer cells. Representative images show that significant DNA damage occurs after 3,5-DMAP exposure in A549 cells. NAC treatment markedly reduced DNA damage caused by 3,5-DMAP. Previously, we have determined that exposure to 25 μM of 3,5-DMAP caused increase in DNA damage in both CHO AS52 cells and AA8 cells and this damage was prevented by both ascorbic acid and selenocompounds [27]. Therefore, we can postulate that high intracellular ROS levels lead to oxidative DNA damage, which can further result in mutations and possibly cancer. Generally, oxidative DNA damage is repaired by multiple pathways, including base excision repair (BER) [35], nucleotide excision repair (NER) [36], and nucleotide incision repair [37]. However, in the previous work [24], we did not observe any of different DNA damage responses between NER-proficient and NER-deficient CHO cells, suggesting that the repair pathways other than NER, might be active in reducing 3,5-DMAP-induced DNA damage.

### *3*.*3* Induction of apoptosis-related proteins and cell cycle regulator proteins

**Fig 3:**
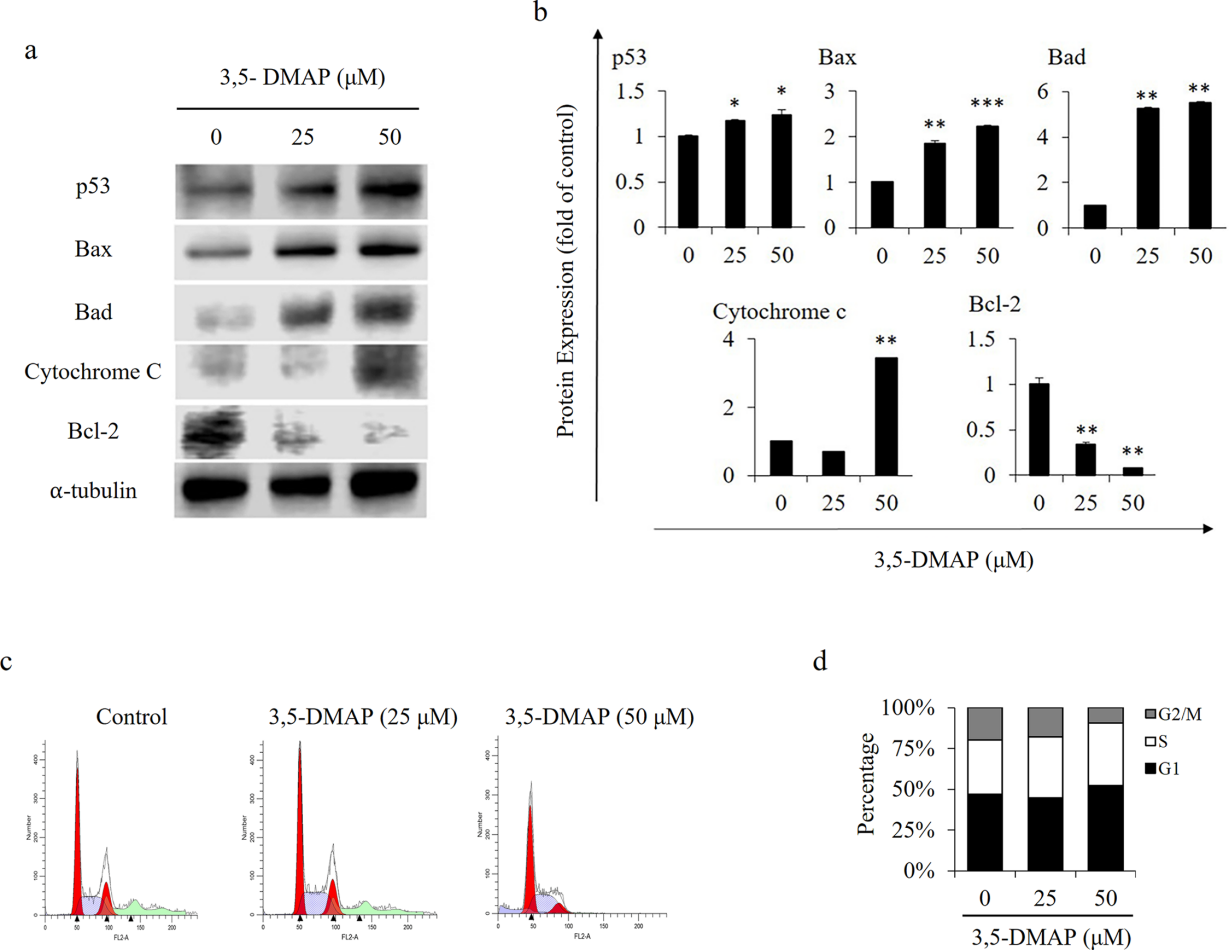
*3*,*5-DMAP* caused both activation of apoptosis and cell cycle arrest. (A) The expression of apoptotic marker proteins p53, Bax, Bad and cytochrome c, and anti-apoptotic marker Bcl-2 were determined by Western blotting. α–tubulin was used as the internal control. (B) Quantification of p53, Bax, Bad and cytochrome c and Bcl-2 as the relative change of the control. (C) Cell cycle analysis. The values represent the number of cells at various phases of cell cycle as a likely percentage of total cells. (D) Quantification analysis of G1, S, and G2/M cell cycle from flow cytometry. Cell cycle percentages were derived from flow cytometric analysis of total cell number in each group.

Upon DNA damage, p53 accumulates immediately through a post-transcriptional process that leads to apoptosis [38, 39]. Bax and Bad are pro-apoptotic proteins which trigger mitochondrial membrane disruption [40]. Bcl-2 plays a vital role in cell survival in response to apoptotic stimuli through inhibition of mitochondrial cytochrome c release [41]. As shown in Fig 3A, Bax, Bad, cytochrome c and p53 protein levels are significantly increased, while Bcl-2 protein expression was decreased in a dose dependent manner. These results demonstrate apoptosis can be induced after 1 h exposure to 3,5-DMAP. Moreover, 3,5-DMAP exposure caused higher p53 expression with increasing doses. This shows that this particular tumor suppressor protein accumulates immediately and causes cell growth arrest in a dose-dependent manner [42, 43]. Bax is reported to interact with and increase the opening of mitochondrial voltage-dependent anion channel (VDAC), which in turn leads to the loss in membrane potential and the release of cytochrome c [44]. On the other hand, Bad protein, a pro-apoptotic member of the Bcl-2 gene family, can positively regulate cell apoptosis by forming heterodimers with BCL-xL and BCL-2, and reverse their death repressor activity. The increase in the expression of Bax and Bad proteins is regulated by p53 protein and has been shown to be involved in p53-mediated apoptosis. Therefore, we can suggest that 3,5-DMAP exposure leads to increase in p53 exposure which later causes increases in Bax expression and this phenomenon is also dose-dependent.

As 3,5-DMAP was found to cause cytotoxic effects in different CHO cells [28] and also in A549 lung cells, we examined whether this chemical could alter the ability of A549 lung cells to progress through the cell cycle. The DNA content of A549 cells at G2/M significantly reduced from 20% to 9.4% at 50 μM of 3,5-DMAP vs. control (p<0.01) (Fig 3C). The percentage of cells in the G1 phase increased from 46% to 52%. However, the cells were unaffected from 25 μM treatment of 3,5-DMAP. These alterations revealed a G2/M phase shortage and G1 phase arrest (Fig 3D). Previously, we observed that 3,5-DMAP treatment at 25 μM dose caused decrease in the percentage of cells that were in S and G2/M phases. Moreover, we have also observed that 3,5-DMAP caused G1 phase arrest in CHO AA8 cells. Ascorbic acid mitigated the 3,5-DMAP induced cell cycle arrest and raised the cell count in S and G2/M phases [28].

### *3*.*4 ROS* generated by *3*,*5-DMAP* reduced cell viability and induced *caspase 3* activity, late apoptosis and necrosis

**Fig 4:**
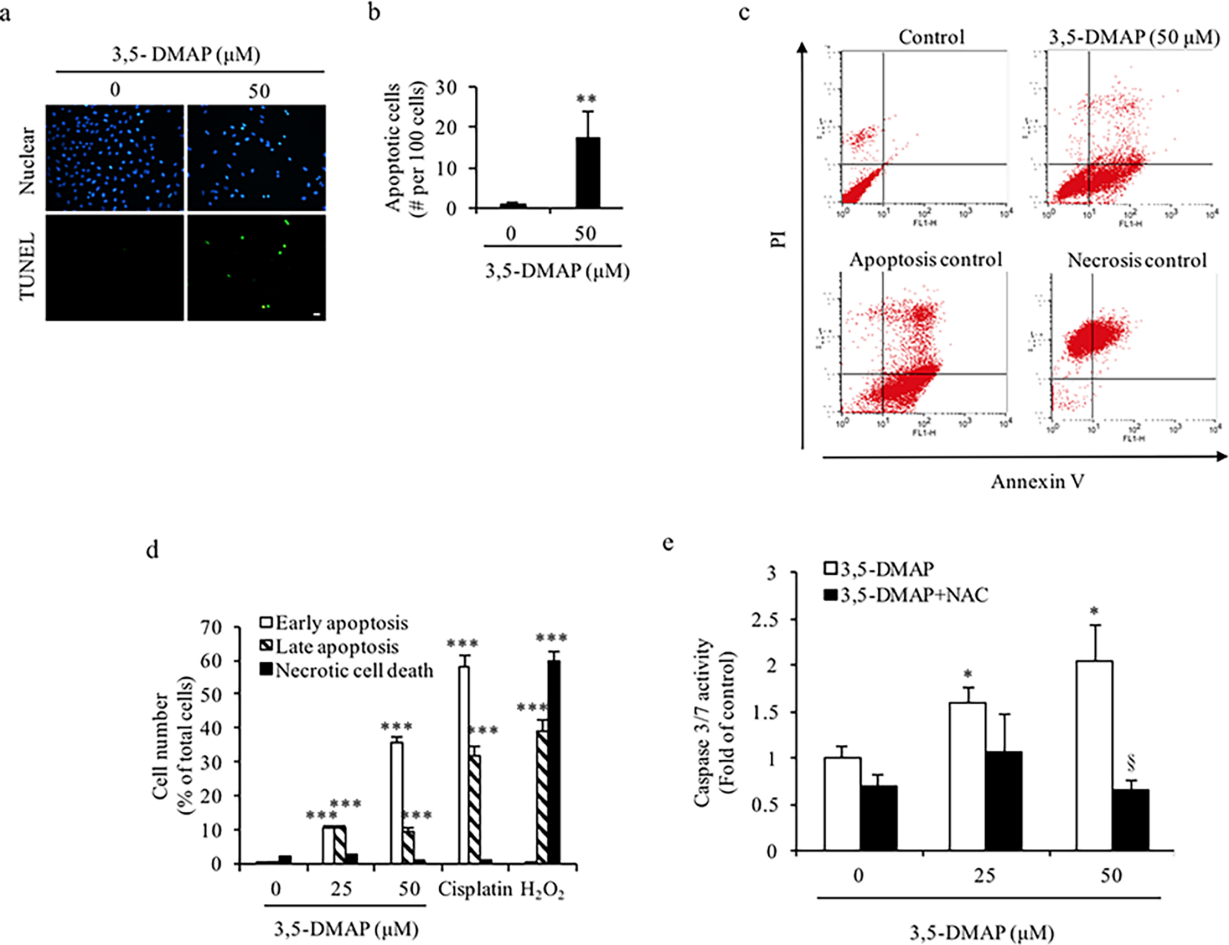
3,5-DMAP induced caspase 3-mediated apoptosis in A549 cells. (A) Apoptosis detection was performed using the TUNEL assay, which was visualized with immunofluorescence microscopy 100X magnifications (Olympus IX51 Inverted Microscope) with emission at 495–529 nm. Scale bar = 50 μm. Nuclei were stained with DAPI, shown in the upper panel. Green spots in the lower panel present apoptotic cells. The cells treated with cisplatin (25 μM) and H_2_O_2_ (100 μM) are the positive controls for apoptosis and necrosis, respectively. (B) Quantitation of TUNEL assay. Values were divided by the amount of nuclei stain in the assessed region and expressed as the number of apoptosis events per 100 cells. (C) Apoptotic cells were identified by flow cytometric analysis using Annexin V-FITC and PI staining. (D) Dose-responses based on all data are plotted. (E) 3,5-DMAP-induced ROS caused caspase 3/7 activity. § shows p<0.01 compared with 3,5-DMAP-treated samples.

After 3,5-DMAP exposure, ROS production increased in a dose-dependent manner (Fig 2B). Generally, apoptosis is virtually the sole mechanism responsible for loss of viability, and the principal mechanism of toxicity against oxidative insults in mammalian cells [45]. To characterize further mechanisms 3,5-DMAP-induced cell death, apoptotic cells were identified by TUNEL assay and flow cytometry after Annexin V-FITC and PI staining and caspase 3/7 method. As shown in Fig 4A, 50 μM 3,5-DMAP exposure caused increases in TUNEL-stained cells, indicating an increase in apoptosis (green spots). When NAC was applied to the cells, the apoptosis caused by 3,5-DMAP (50 μM) declined significantly. Fig 4B shows the quantification of apoptotic A549 cells (assessed from Fig 4A) by counting the number of green spots, dividing by the number of nuclei in the same field, and normalizing the quotient to per 100 cells. In the untreated control, 1.9% of the cells underwent apoptosis. 3,5-DMAP treatment (50 μM) caused a significant increase in apoptotic cell count (17.5%). There was a significant increase in late cell apoptosis after 3,5-DMAP exposure when compared to control; however it was not dose-dependent (Fig 4D). 3,5-DMAP (25 μM) exposure caused 20.7% of A549 cells to undergo apoptosis after 24 h. When cells in early and late apoptosis are combined, 3,5-DMAP (50 μM) caused marked increases in apoptosis, with apoptotic cells reaching to nearly 46% of the total cell count. Cisplatin, a well-known chemotherapeutic agent was used as the positive control (Fig 4D). Flow cytometric analysis revealed that 3,5-DMAP (50 μM) also increased necrotic cell death. Furthermore, Fig 4E shows 3,5-DMAP exposure upregulated caspase 3/7 activity in A549 cells while NAC treatment with 3,5-DMAP protected the A549 cells against 3,5-DMAP induced apoptosis.

These results demonstrated that 3,5-DMAP induced oxidative stress, which in turn led to the induction of apoptosis. Previous studies showed that 3,5-DMAP, like other oxidant chemicals, is able to induce cytotoxicity by causing ROS production, DNA damage and apoptosis in an *in vitro* model [46, 47]. We suggest that 3,5-DMAP has high apoptosis-inducing potential and can cause apoptosis or necrosis in A549 lung cancer cells.

### *3*.*5 3*,*5-DMAP* reduces carcinogenic properties of *A549* cells in vitro and in vivo

**Fig 5:**
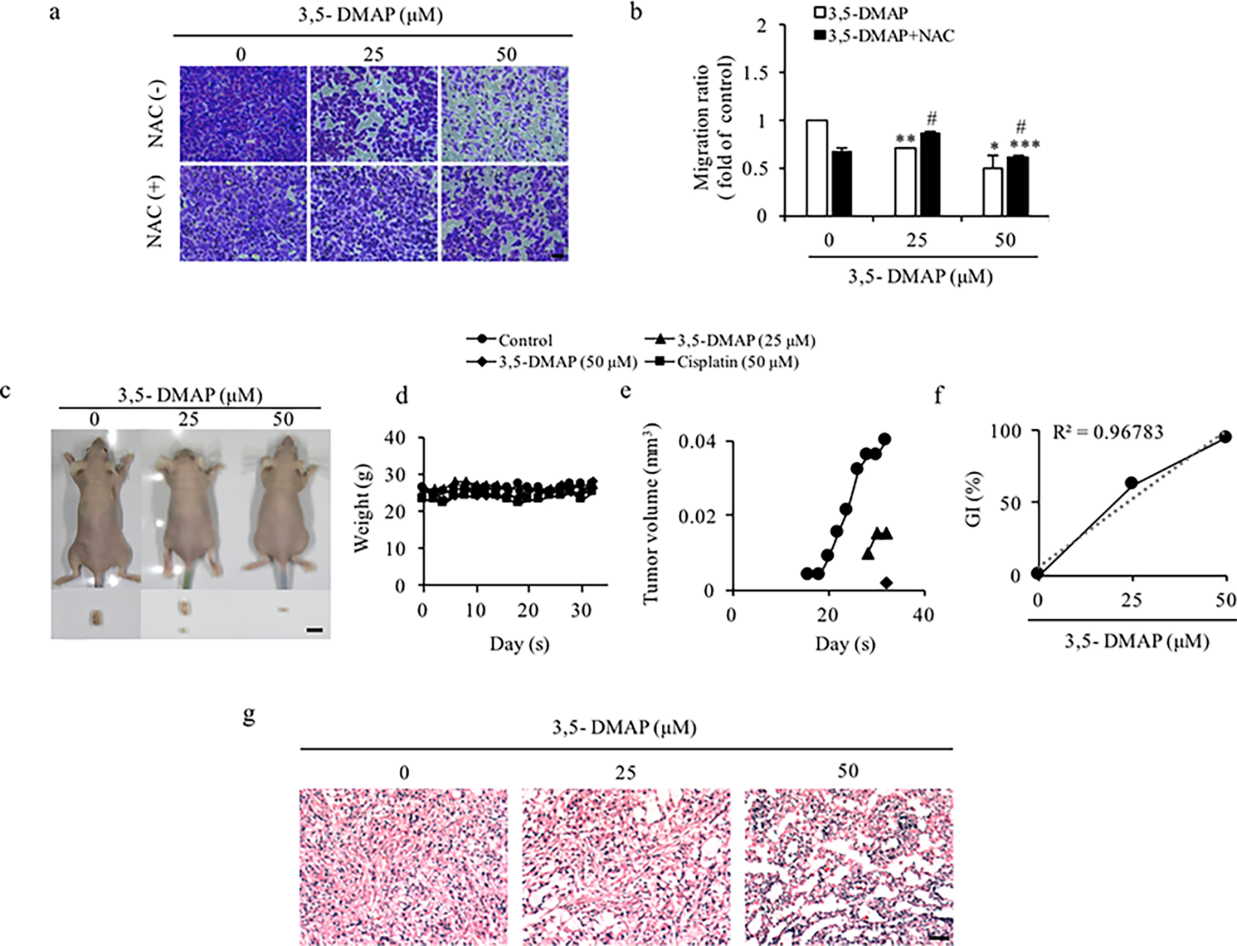
3,5-DMAP exposure decline the carcinogenic property of A549 lung cells. (A) The migrated cells were visualized with using a light microscope with 200 × magnification. Scale bar = 50 μm. (B) Values were presented as the fold change of the control. (C) Two million of cells were injected subcutaneously into the nude mice. The down panel shows the representative subcutaneous flank tumor in the mice. Scale bar = 1 cm. (D) Body weight is indicated by plotted and it shows no difference between the samples. (E) Plots of tumor volumes (cm^3^) determined by measurements with a caliper. (F) Plots of Growth Inhibition (GI) were calculated by tumor volumes. (G) The 4 μm tumor sections were examined by H&E staining. Magnification = 200×. Scale bar = 50 μm. # shows p<0.05 compared with 3,5-DMAP-treated cells.

Epithelial-mesenchymal transition (EMT) is a process in which epithelial cells lose their polarity and ability to adhere. Instead, they gain properties to move, migrate through the extracellular matrix and become invasive [48]. It is well known that cancer cells possess a very high capacity of migration. Metastasis is a crucial step for the cancer cells. It causes cancer cells to spread to different tissues, and organs [49, 50]. In order to examine whether the migration capacity of A549 cells can be altered by 3,5-DMAP (50 μM), we performed “cell migration assay”. We observed that 3,5-DMAP suppressed the migration ability of A549 cells. As shown in Fig 5A, we found the amount of cells inserted on the bottom membrane of trans-well decreased dose-dependently. The quantitation data shows that 25 and 50 μM of 3,5-DMAP significantly reduced the migration ratio from 1 to 0.70 and 0.5, respectively (Fig 5B). Addition of NAC with 50 μM of 3,5-DMAP also significantly suppressed migration capacity of the cells when compared to untreated controls. This finding suggests that 3,5-DMAP has anti-metastatic potential by decreasing the migration capacity of A549 cells.

In an *in vivo* model, we also assessed whether 3,5-DMAP attenuated cancer cell growth or not. After 32 days of observation, we found that 3,5-DMAP could inhibit tumor growth (Fig 5C–5E). The percentage of growth inhibition reveals that 25 μM 3,5-DMAP inhibited tumor growth by 62.5% and 50 μM 3,5-DMAP inhibited tumor growth by 95% after 32 days (Fig 5F). In the positive control, cisplatin treated sample inhibited tumor growth reach almost 100%. The hematoxylin & eosin staining (Fig 5G) showed that tumor was damaged by 3,5-DMAP exposure. In addition, tissue sections displayed loose and appeared vacant particularly at the highest dosing level and the tumor nearly disappeared after 3,5-DMAP treatment.

### *3*.*6* Immunohistochemistry staining indicates that *3*,*5-DAMP* attenuates tumor growth in vivo

**Fig 6:**
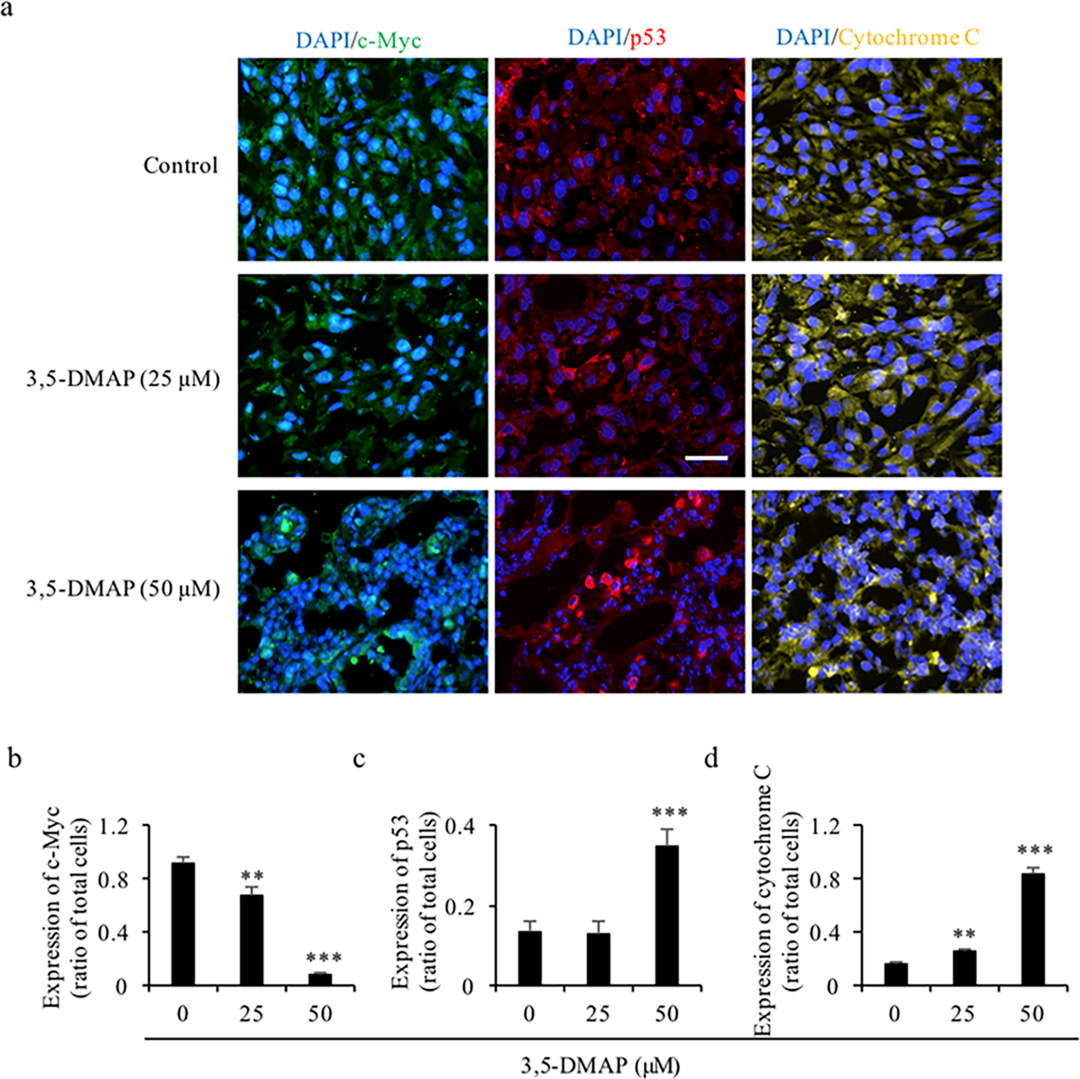
3,5-DMAP exposure down-regulates the expression of oncogene c-Myc and induces apoptosis. (A) The 4 μm tumor sections were examined by c-Myc, p53 and cytochrome c immunohistochemistry staining. Magnification = 200×. Scale bar = 25 μm. (B) Quantitative analysis of c-myc expression. (C) Quantitative analysis of p53 expression. (D) Quantitative analysis of cytochrome c expression.

c-Myc is an oncogene, high expressions of which can promote tumor formation. Immunostaining was used to detect c-Myc expression. As shown in Fig 6A, c-Myc was significantly decreased after 25 and 50 μM 3,5-DMAP treatments in A549 lung cells. The expression rate of c-Myc dropped to 0.08 fold of control after application of 50 μM of 3,5-DMAP (Fig 6B). Comparing to Fig 3 of *in vitro* data, we also examined *in vivo* apoptotic p53 and cytochrome c expression rates, also by immunostaining. The results showed that both p53 and cytochrome c expression rates increased after exposure to 3,5-DMAP in a dose dependent manner (Fig 6A–6D). Thus, in A549 cells, 3,5-DMAP induced apoptosis through the higher expression of p53, by halting of cell cycle and through higher expression of apoptotic pathway proteins. Together, these findings indicate that 3,5-DMAP exposure could attenuate cancer cell transformation and therefore could stop or delay tumor formation.

## 4. Conclusion

**Fig 7:**
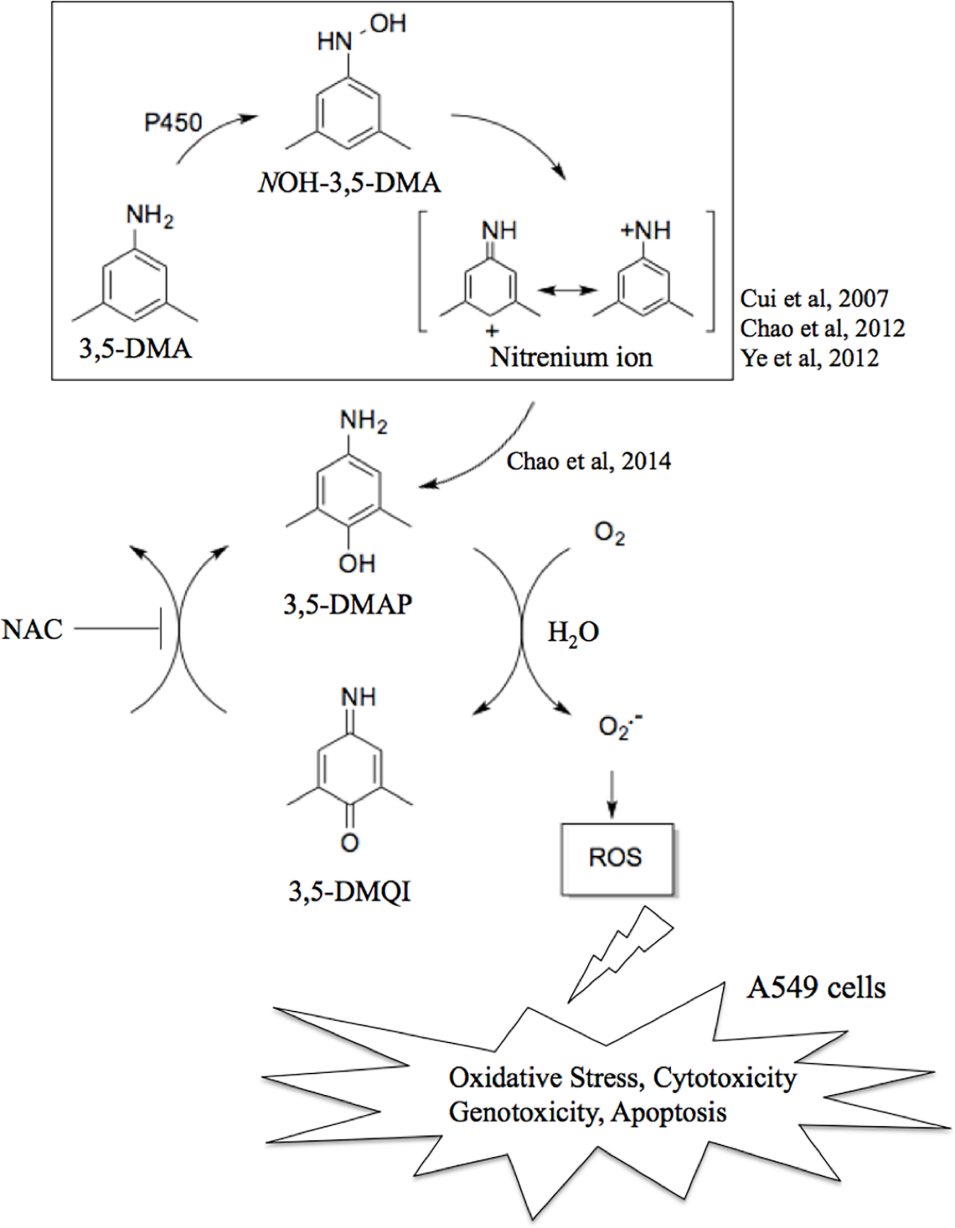
Pathway for production of ROS by 3,5-DMAP. 3,5-DMA undergoes biotransformation with N–O bond heterolysis and converts to an unstable *N-*OH-3,5-DMA and highly reactive intermediate nitrenium ion, which is able to react with a DNA base to produce a mutagenic adduct [25]. 3,5-DMAP can be produced by cytochrome P450-catalyzed hydroxylation of 3,5-DMA or by nucleophilic attack of H_2_O on the appropriate resonance form of the nitrenium ion. 3,5-DMAP readily undergoes redox cycling mechanism and converts to 3,5-dimethylquinoneimine (3,5-DMQI). The process accompanies generation of superoxide ion (O_2_^.-^). NAC can attenuate this cycling.

3,5-DMAP causes ROS production in non-small cell lung cancer A549 cells (Fig 7). The intracellular oxidation state can be induced by the particular product, 3,5-DMQI, in the redox cycle to resulting in both cytotoxicity and genotoxicity in cancer cells. Therefore, we suggest that 3,5-DMAP induces an imbalance in cellular antioxidant/oxidant status and causes both lipid peroxidation and DNA damage. This phenomenon can further trigger molecular events, which might eventually cause alterations in tumor cell cycle and initiate of apoptosis. In addition, 3,5-DMAP is able to suppress the migration ability of A549 cells. Moreover, 3,5-DMAP could inhibit tumor growth and reduce the size of tumors *in vivo*. These findings suggest that 3,5-DMAP or its derivatives might be anti-cancer drug candidates. On the other hand, NAC is found to be protective against the oxidative stress and is able to reduce both cytotoxicity and genotoxicity induced by 3,5-DMAP exposure in A549 lung cells. Therefore, we suggest that if people who are smoking or exposed to second hand smoke (therefore exposed to 3,5-DMA) have stable blood levels of NAC and/or glutathione, they might be protected against the toxic effects of this particular alkylaniline. However, further experiments (particularly on anti-cancer effect—structure relationship) are needed to show the anti-carcinogenic effects of 3,5-DMAP and whether 3,5-DMAP or its analogues are promising agents for the treatment of different types of cancers, particularly for cancer of the lung.
